# Automatic Treatment Planning for Radiation Therapy: A Cross-Modality and Protocol Study

**DOI:** 10.1016/j.adro.2024.101649

**Published:** 2024-10-09

**Authors:** Gregory Szalkowski, Xuanang Xu, Shiva Das, Pew-Thian Yap, Jun Lian

**Affiliations:** a Department of Radiation Oncology, University of North Carolina, Chapel Hill, North Carolina; b Department of Radiation Oncology, Stanford University, Stanford, California; c Department of Radiology and Biomedical Research Imaging Center, University of North Carolina, Chapel Hill, North Carolina

## Abstract

**Purpose:**

This study investigated the applicability of 3-dimensional dose predictions from a model trained on one modality to a cross-modality automated planning workflow. Additionally, we explore the impact of integrating a multicriteria optimizer (MCO) on adapting predictions to different clinical preferences.

**Methods and Materials:**

Using a previously created 3-stage U-Net in-house model trained on the 2020 American Association of Physicists in Medicine OpenKBP challenge data set (340 head and neck plans, all planned using 9-field static intensity modulated radiation therapy [IMRT]), we retrospectively generated dose predictions for 20 patients. These dose predictions were, in turn, used to generate deliverable IMRT, VMAT, and tomotherapy plans using the fallback plan functionality in Raystation. The deliverable plans were evaluated against the dose predictions based on primary clinical goals. A new set of plans was also generated using MCO-based optimization with predicted dose values as constraints. Delivery QA was performed on a subset of the plans to assure clinical deliverability.

**Results:**

The mimicking approach accurately replicated the predicted dose distributions across different modalities, with slight deviations in the spinal cord and external contour maximum doses. MCO optimization significantly reduced doses to organs at risk, which were prioritized by our institution while maintaining target coverage. All tested plans met clinical deliverability standards, evidenced by a gamma analysis passing rate >98%.

**Conclusions:**

Our findings show that a model trained only on IMRT plans can effectively contribute to planning across various modalities. Additionally, integrating predictions as constraints in an MCO-based workflow, rather than direct dose mimicking, enables a flexible, warm-start approach for treatment planning, although the benefit is reduced when the training set differs significantly from an institution's preference. Together, these approaches have the potential to significantly decrease plan turnaround time and quality variance, both at high-resource medical centers that can train in-house models and smaller centers that can adapt a model from another institution with minimal effort.

## Introduction

In the treatment of cancer, radiation therapy (RT) serves as a valuable tool and is estimated to provide a benefit for about half of all patients with cancer.^1^ For head and neck cancers, RT is indicated for an even higher percentage (approximately 74%) of the patient population.^2^ Although many clinical trials have established suggested prescription doses as well as healthy tissue dose constraints for the treatment of these cancers,^3^, ^4^, ^5^ it may not always be possible to meet all these objectives for a given patient. In these cases, the planner and the physician must determine what trade-offs should be made to provide the best outcome for the patient.

In standard planning, the planner will set several optimization parameters, consisting of some dose objective and associated weight that the treatment planning system (TPS) then uses to create an optimized plan. Because of variations in patient anatomy and treatment intent, the “ideal” optimization parameters are different for each case, and the planner must iteratively adjust the parameters to try to improve the plan. This is complicated by the fact that the relationship between adjustments to the optimization parameters and the resulting changes to the optimized treatment plan is not always intuitive,^6^^,^^7^ resulting in a labor-intensive process, especially for planners with less experience. This means that the quality of the final treatment plans is often dependent on the planner's experience and available time for the planning process,^8^, ^9^, ^10^ and low-quality plans can lead to worse clinical outcomes for patients.^11^^,^^12^

The use of automation can help with both reducing this variability in plan quality as well as helping increase patient throughput by speeding up the typically slow trial and error planning process.^13^^,^^14^ A variety of auto-planning methods have been developed,^15^^,^^16^ which can be roughly split into (1) knowledge-based planning, such as dose-volume histogram (DVH) guidance, in which DVHs for contoured structures are predicted based on anatomical and geometric features,^17^^,^^18^ (2) protocol-based optimization, in which changes to the optimization parameters are automatically implemented to minimize organ at risk (OAR) dose while meeting user defied clinical constraints,^19^^,^^20^ (3) automated multicriteria optimization, in which the software generates a set of parieto-optimal plans and allows for either the user or the software itself to select the “best” solution based on the treatment site and clinical protocol,^21^, ^22^, ^23^ and, most recently, (4) statistical models, including machine learning, which attempt to learn the correlation between patient anatomy and the resulting plan.^24^^,^^25^ Recent approaches have used deep learning for dose prediction,^26^, ^27^, ^28^, ^29^ which could then be used to generate a plan via dose-mimicking approximation or by directly predicting the fluence map that would produce the desired dose distribution.^30^^,^^31^

**Figure 1:**
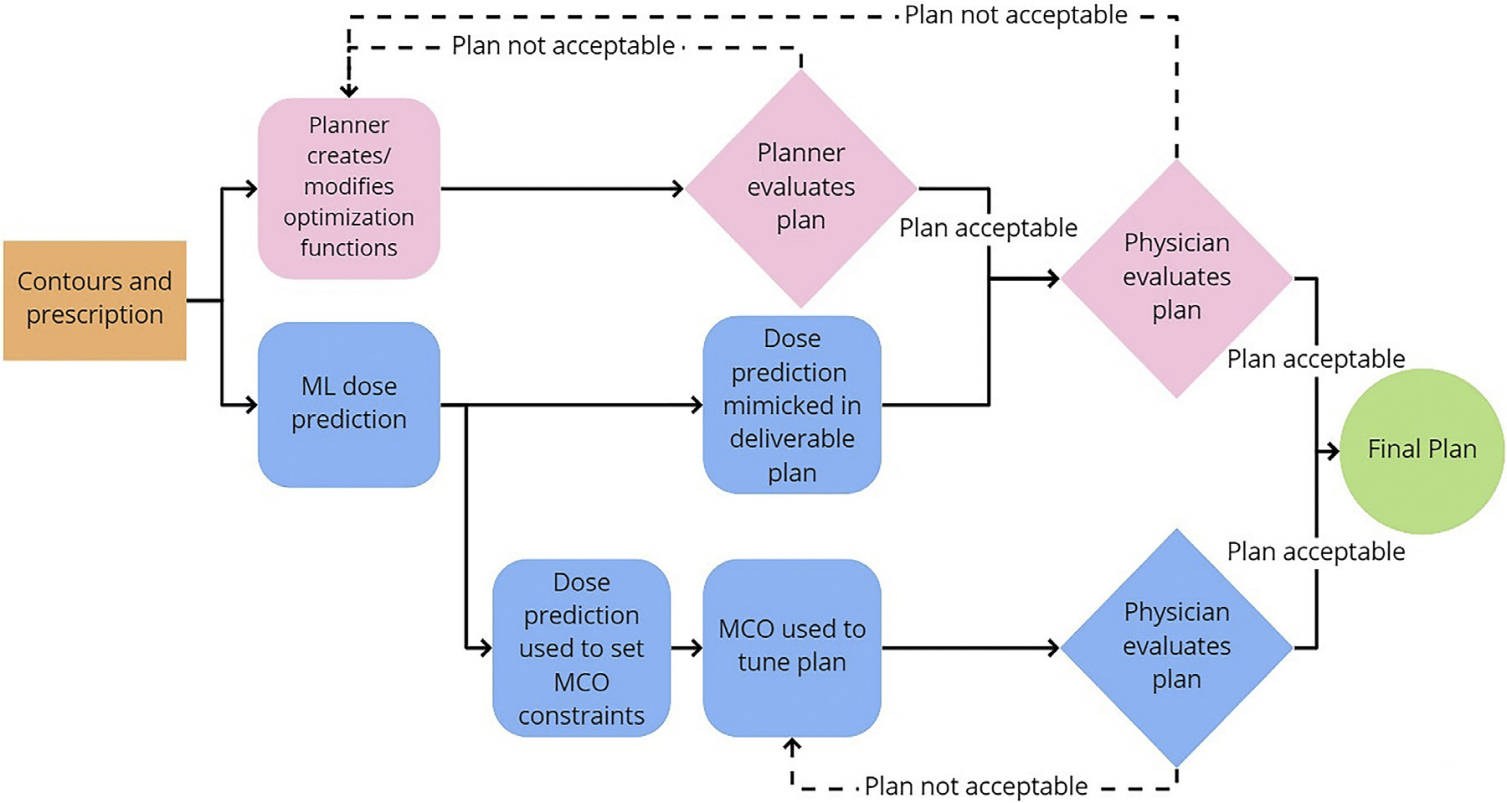
Conventional and proposed workflows. The conventional workflow (top, pink) requires a great deal of manual work, with the dosimetrist manually iterating through many possible plans. Our proposed workflows (bottom, blue) significantly cut down on this work by providing a prediction of what the dose distribution for a given patient would look like based on prior plans. Although the mimic workflow may require some further optimization by the planner, the expected number of iterations is much less than in the conventional workflow. *Abbreviations:* MCO = multicriteria optimizer.

In this work, we proposed 2 automatic treatment planning methods, shown in Fig. 1, a mimicking approach and a multicriteria optimizer (MCO) approach. For the mimicking approach, we evaluate the applicability of the predicted dose distribution generated by our previously developed deep learning model^32^ to the achievable dose distribution using the same delivery modality as the model training set, fixed-gantry intensity modulated RT (FG-IMRT), as well as different delivery modalities, volumetric modulated arc therapy (VMAT) and tomotherapy. By using the fallback planning module to do the optimization, instead of iteratively adjusting a set of optimization objectives, plan generation requires less input from the dosimetrist and can increase the efficiency of the planning process. Because the model will output a predicted dose distribution similar to the plans used for training, this approach is less suitable if a large variety of protocols, the directives stating the prescription dose to the target(s) and dose constraints of healthy tissue structures, are to be used. For instance, if the training plans are from an institution that prioritizes minimizing cord dose, but the institution that is using the model is following a protocol that prioritizes lowering the parotid dose but allows for a higher cord dose, the predicted dose distribution may not fully align with the desired treatment plan. To address this, we investigated if integrating the MCO into our workflow allows for modifications to be made to the plan dose distribution to better conform to institutional or individual physician preference without requiring the creation of a new model.

## Methods and Materials

### Model creation

**Figure 2:**
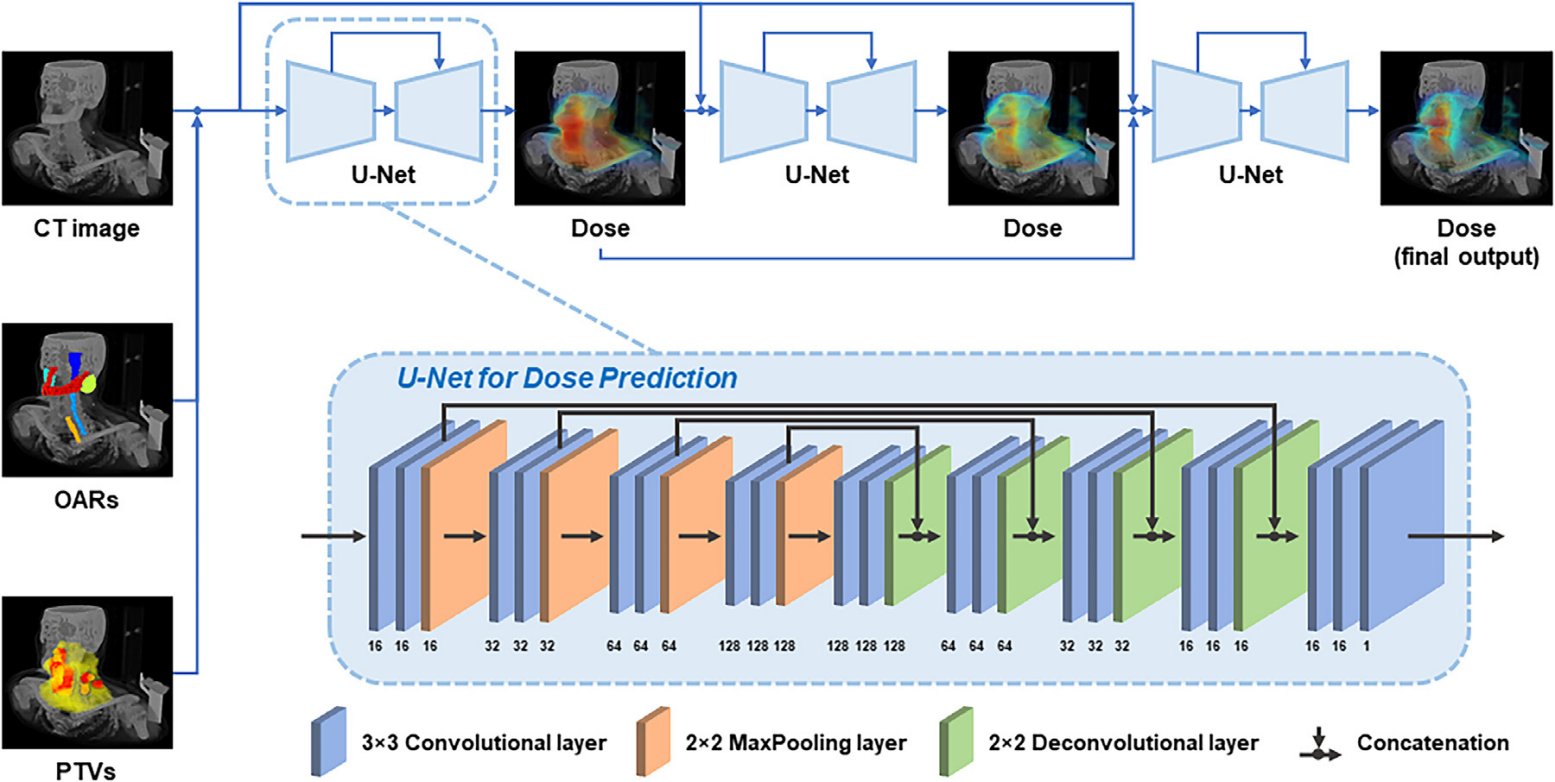
Schematic diagram of the network architecture of our dose prediction model. The bottom subfigure illustrates the detailed structure of each 3-dimensional U-Net used for dose prediction. The numbers annotated below the layers indicate their output channel number. *Abbreviations:* CT = computed tomography; OAR = organ at risk; PTV = planning target volume.

Our dose prediction model^32^ uses a triple-stage cascaded U-Net architecture to predict the dose distribution based on the input computed tomography (CT) image and the planning target volume (PTV)/OAR structure set. The schematic diagram of our model is illustrated in Fig. 2. This model consists of 3 cascaded U-Nets, each of which follows the original U-Net architecture in Ronneberger et al^33^ (except for the input and output layers modified to adapt to our dose prediction task), sequentially predicting the dose-volume in a coarse to fine manner using the auto-context mechanism.^34^ Specifically, the first U-Net takes the CT image concatenated with the N region of interest (ROI) (OAR and PTV) binary masks as a (N + 1)-channel input and outputs a single-channel coarse dose volume. This coarse dose volume is then concatenated with the CT image, and the N ROI binary masks as a (N + 2)-channel input for the second U-Net to predict another single-channel dose volume that is more accurate than the first one. Finally, the third U-Net takes the 2 dose volumes predicted by the first and second U-Net concatenated with the CT image, and the N ROI binary masks as a (N + 3)-channel as input to generate the final dose volume, which is expected to be a refinement of the previous 2 dose volumes. The 3 U-Nets share identical network structures (which follow the original U-Net architecture in Xu et al^32^ and are illustrated at the bottom of Fig. 2 in detail) except for the input channel numbers of the first convolutional layer, which are N + 1, N + 2, and N + 3, respectively. The output channel number of the last convolutional layer of these 3 U-Nets is set to be 1, representing the predicted single-channel dose volume. Our network is fully implemented in a 3-dimensional (3D) manner using 3D convolutional layers and pooling layers (which means it takes the 3D CT volume as input, not 2-dimensional CT slices). During both the training and testing phase, we normalized the CT values from (−600 to 1400) HU to (0-1) and the dose values from (0-80) Gy to (0-1). In the training phase, we trained the model using the Adam optimizer with a base learning rate of 0.0005 for 400 epochs (approximately 17 hours) with a batch size of 6. The training procedure was conducted on a server computer equipped with 2 Intel(R) Xeon(R) E5-2650 CPUs working at 2.20 GHz and 6 NVIDIA TITAN Xp graphic cards with 12 GB of memory each. We implemented our model using the PyTorch framework. After training, the model can generate a prediction for a new case in approximately 1 to 2 seconds.

We used the data set provided for the 2020 American Association of Physicists in Medicine OpenKBP (Knowdege Based Planning) challenge,^35^^,^^36^ consisting of 340 head and neck plans, which all used 9-field IMRT. Each plan had either just a high-risk PTV, a high-risk and a low-risk PTV, or a high-, intermediate-, and low-risk PTV. The dose prescription for the PTVs was 70 Gy, 63 Gy, and 56 Gy for the high-, intermediate-, and low-risk volumes, respectively. Two hundred plans were used for training, 40 for validation, and 100 for testing. The model placed fifth in the DVH assessment and eighth in the dose assessment out of 195 registered participants, according to the result on the official challenge closing date of May 29, 2020. The results of the top contestants can be seen in Table 1.

**Table 1:** Top 10 results from the American Association of Physicists in Medicine OpenKBP challenge

Dose score (rank)	DVH score (rank)
2.4294 (1)	1.4776 (1)
2.5635 (2)	1.7045 (12)
2.6146 (3)	1.5818 (7)
2.6497 (4)	1.5393 (2)
2.6795 (5)	1.5728 (6)
2.7449 (6)	1.7408 (14)
2.7477 (7)	1.7055 (13)
**2.7528 (8)**	**1.5559 (5)**
2.7786 (9)	1.5515 (3)
2.8137 (10)	1.5549 (4)

Results for the model used in this work are highlighted (eighth in dose prediction and fifth in dose-volume histogram prediction). The scores are roughly the mean absolute error for the dose and target/organ at risk dose-volume histograms, respectively. Details on the scoring can be found in Babier et al.^35^

### Same modality assessment

After generating the dose predictions using our machine learning model, we imported the predicted dose, CT, and RT structure data for 20 patients into our TPS, Raystation (RaySearch Laboratories). We then set up a plan template with the same beam arrangement as the plans used in the training set: 9 IMRT beams from 0 to 320°, with 40° between each beam. The beam isocenter was set using the ballbearings (BBs) placed on the patient's skin, if available, or at the center of the high-risk PTV. We then set the beam optimization parameters to our institutional standard to ensure the resulting plans would be delivered to our machines. For IMRT plans, the minimum segment area was restricted to 4 cm^2^, the minimum segment monitor unit (MU) per fraction was set to 5 MU, and the minimum leaf end separation was set to 1 cm.

Using Raystation's mimic dose functionality, we optimized the plan for each patient to try to match the dose prediction. For each case, the optimization was run for 180 iterations or until convergence, whichever happened first. All the plans were normalized to give 95% coverage at 70 Gy to the high-risk PTV, per our institutional standard. The resulting plan dose was then compared against the predicted dose using the methods laid out in section 2.6.

### Cross-modality: FG-IMRT to VMAT

To assess if the predicted dose could be extended to treatment modalities other than those used in the plans in the training set, we also applied the mimic dose technique to create 2 arc VMAT plans, which is the primary modality used at our clinic. For the VMAT plans, the leaf motion was constrained to 0.48 cm/degree.

Although the plan template was different in these cases, the rest of the process was the same as the modality plans; a deliverable plan was optimized using the mimic dose functionality and the predicted dose distribution as the reference.

### Cross-treatment machine: FG-IMRT to tomotherapy

Finally, we also assessed if the predicted dose could be extended entirely to a different delivery device, a tomotherapy unit. For the tomotherapy plans, we used a dynamic field width of 2.5 cm, a pitch factor of 0.303, and a delivery time factor maximum of 1.5, the standard settings used for head and neck planning at our institution.

### Dosimetric protocol

To allow for more customization of the plan to conform to differences in preference between institutions and individual physicians and to help improve plan quality when the prediction is not optimal, we then used the dose prediction to inform the use of the MCO to create deliverable plans. In brief, for a given set of optimization objectives, the MCO generates a database of Pareto optimal plans that is sampled from the Pareto surface consisting of all possible Pareto optimal plans.^31^^,^^37^, ^38^, ^39^ By navigating this Pareto surface, it is possible to explore a wide range of trade-offs to search for the “best” plan, although in practice, a large area of this surface concerns trade-offs that would be considered clinically unacceptable. By applying constraints set using the knowledge gained from our dose prediction, we can focus the MCO on the area of the Pareto surface containing what would be considered high-quality plans, which can increase the ease of navigation.

In this work, for each case, a standard template of MCO objectives and constraints (Table E1) was loaded, and the constraints were updated to correspond to the results from the dose prediction for the patient. During the MCO optimization, Raystation uses the objectives to create the set of Pareto plans and will only consider Pareto plans that meet all specified constraints. Because we wanted to leave some flexibility for customization and prevent the MCO from becoming overconstrained, we elected to set only some of the predicted clinical goals as constraints. As will be discussed in sections 3.1 to 3.3, the mimic dose functionality had some difficulty replicating the maximum dose to the spinal cord and the external contour, so these were chosen to be set as constraints in the MCO template. We also set the D50 for the parotids as a constraint because, due to their proximity to the target, the parotids were found to have the largest impact on the dose distribution. D50 was used instead of the mean because it was found to be less likely to lead to the MCO optimization becoming overconstrained. Not all contours were present for all patients, namely intermediate-risk PTV, esophagus, and larynx contours, so the associated objective functions were only used when applicable.

After the template was updated with the cases specific constraints from the dose prediction, we ran the MCO optimization using the Raystation recommended parameters, generating a number of Pareto plans equal to twice the number of objectives used (24-30 in these cases, depending on the structures contoured) with a maximum of 40 iterations per Pareto plan. In 2 cases, the maximum dose to external constraint violated the MCO feasibility constraint and was excluded. After the Pareto plans were generated, the “balance plan,” which equally weights all the Pareto plan results, was used without modification to generate the final deliverable plan. In clinical practice, it would be prudent to adjust the weighting of the Pareto plans to improve the dosimetry of more concerned structures and/or conform to institutional preference, but this was not done in this work to maintain a standardized workflow.

To compare against uninformed MCO planning, we then deleted the prediction-informed constraints and reoptimized them using the same procedure described above.

### Comparison metrics

The similarity between the predicted dose and the mimicked or MCO-integrated plans was primarily assessed using the clinical goals established at our institution for the structures that were contoured in the OpenKBP challenge. These metrics consist of the maximum dose to the spinal cord and external, the mean dose to the parotids and larynx, and the D50 to the parotids. Table E2 shows the template goal sheet used across the test cases. The statistical significance of the difference was assessed using a Wilcoxon signed rank test. We defined a dose difference >5% as “clinically relevant.”

### Delivery assessment

To ensure that the mimicked plans were deliverable on the machines, we performed delivery quality assurance tests (DQA) on a selected sample of 5 plans for each modality (IMRT, VMAT, and tomotherapy) for both the mimicked and MCO plans. To do this, we delivered the plans to an ArcCheck device software (Sun Nuclear Corporation) that measured the dose distribution output by the treatment machine. We then compared this measured distribution with the predicted distribution provided by our TPS using a gamma analysis test.^40^

We used 3% for the dose difference, a 2 mm distance to agreement criteria, a low-dose cutoff of 10% of the prescription dose, and considered a plan to be clinically deliverable if ≥90% of points passed, as recommended by the American Association of Physicists in Medicine task group report 218.^41^ The analysis was run using the SNC Patient software provided with the ArcCheck device (Sun Nuclear Corporation).

## Results

### Same modality mimic plans

**Figure 3:**
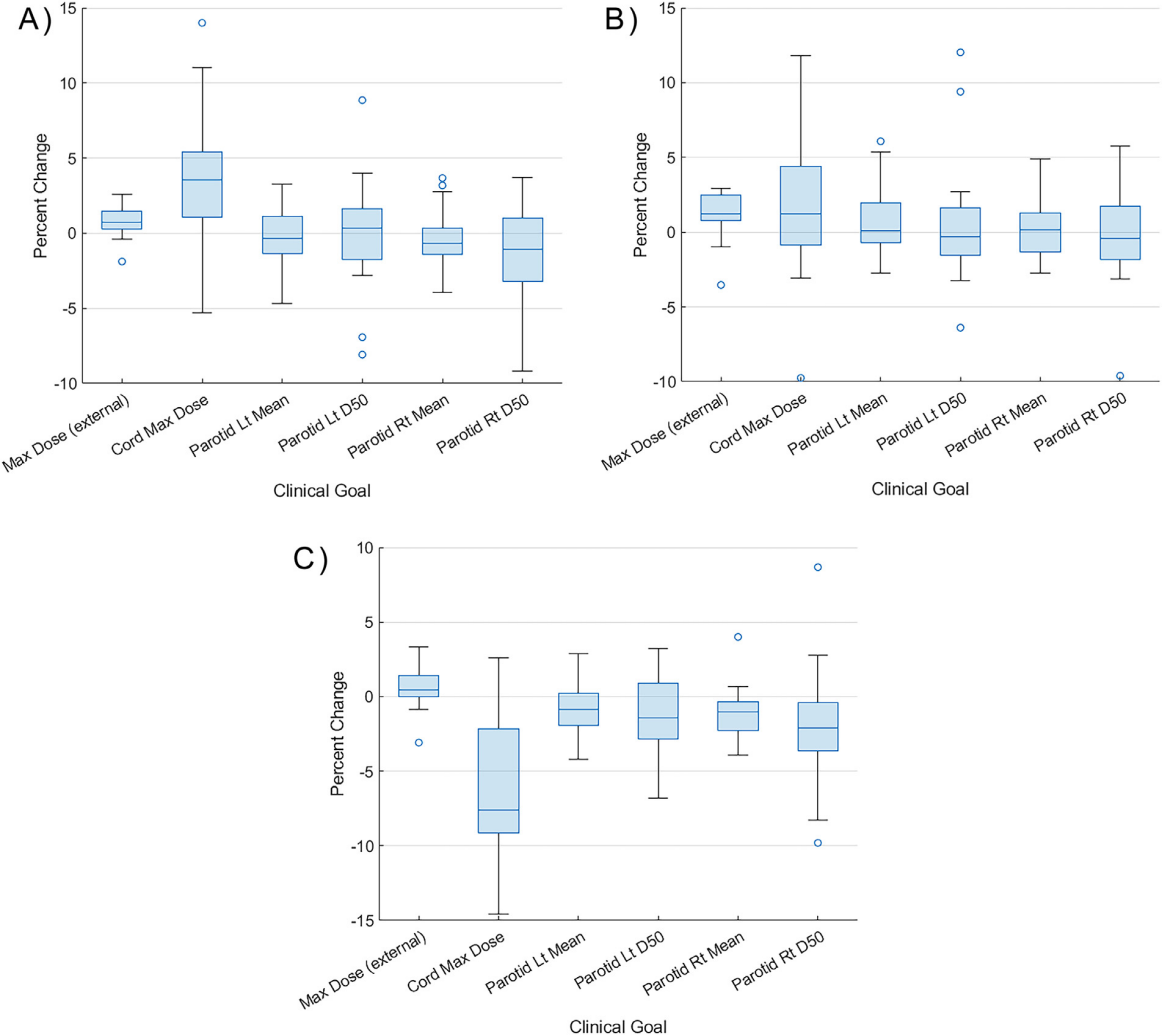
Percent difference of the predicted and mimicked dose distributions. (a) Shows the difference between the prediction and the mimic 9-field intensity modulated radiation therapy plan. The central mark indicates the median, and the bottom and top edges of the box indicate the 25th and 75th percentiles, respectively. Any outliers point >2.7 SDs away from the median and are plotted outside of the whiskers. (b) As in (a) but comparing the dual arc volumetric modulated arc therapy (VMAT) plans. (c) As in (a) but comparing the tomotherapy plans. *Abbreviations:* Lt = Left; Rt = Right; D50 = Dose delivered to 50% of volume.

Except for the spinal cord maximum dose, there was no clinically relevant (>5%) difference between the predicted and mimicked IMRT plans for the relevant dosimetric goals. An extra maximum dose objective on the cord was able to reduce the difference in the cord maximum dose <2% without a significant impact on any of the rest of the clinical goals. As will be covered in the Discussion, the addition of a simple maximum dose objective can assist the mimic workflow in achieving the predicted value for this clinical goal. Figure 3a shows the distribution of the percent difference between the predicted doses and mimicked IMRT plans for select dosimetric values. A full comparison can be found in Table E3. For each case, the prediction-to-plan time was approximately 15 minutes because of the short, standardized workflow (import dose, load plan template, add mimic dose and cord objectives, and optimize).

### Cross-modality from FG-IMRT to VMAT

For the mimic plans created using VMAT, the dosimetry for most organs was still similar to the prediction based on the FG-IMRT model. There were no clinically relevant (>5%) differences for any of the relevant clinical goals, although the difference in the whole body (also known as the external contour) and cord maximum dose was statistically significantly different (*P* < .04) from the prediction. Figure 3b shows the distribution of the percent difference between the predicted doses and mimicked VMAT plans for select dosimetric values. A full comparison can be found in Table E4.

### Cross-modality from FG-IMRT to tomotherapy

**Figure 4:**
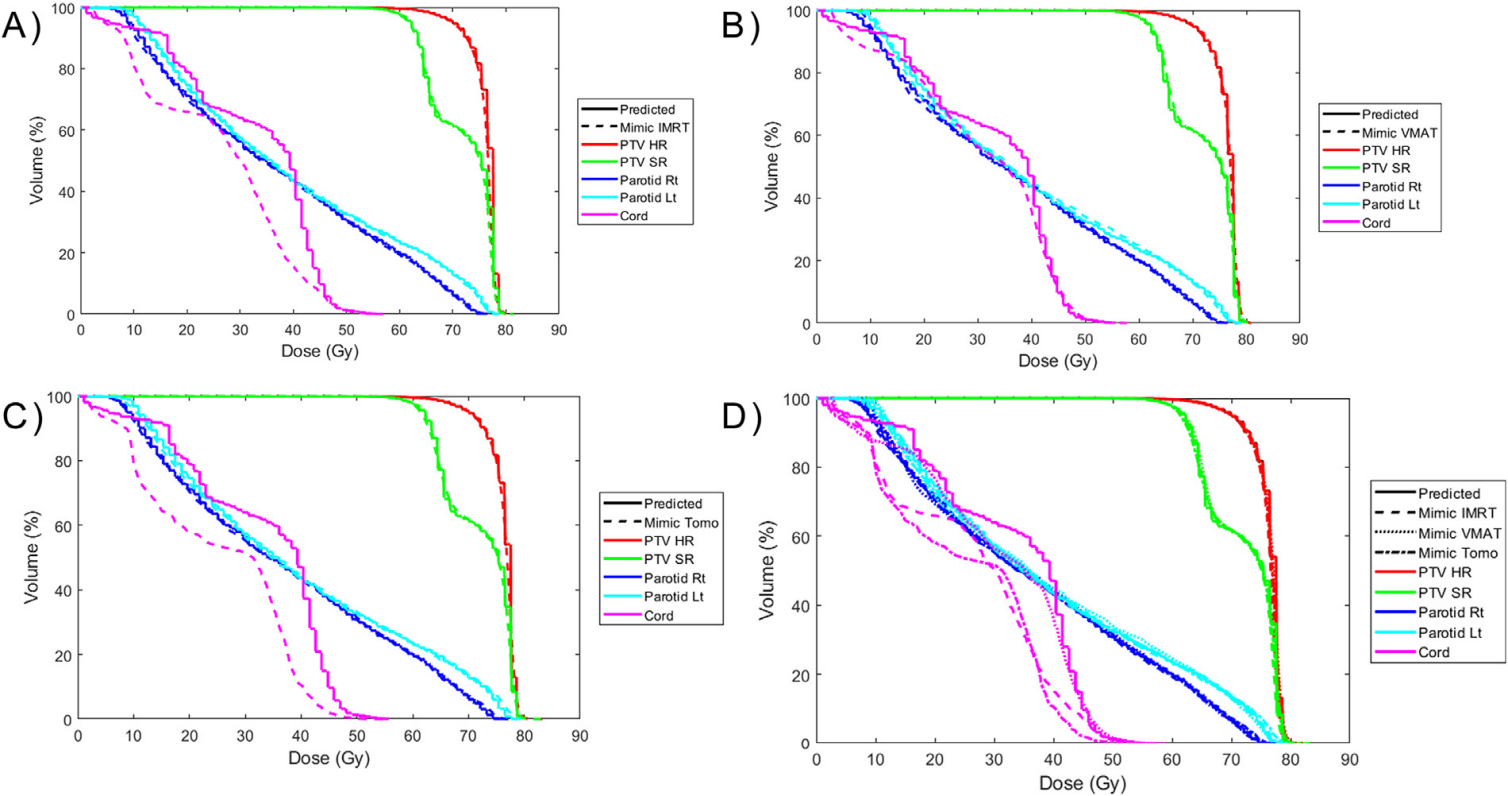
Comparison of the dose-volume histograms for a representative case. The reference is the predicted dose. (a) Comparison with the intensity modulated radiation therapy (IMRT) mimic plan. (b) Comparison with the volumetric modulated arc therapy (VMAT) mimic plan. (c) Comparison with the tomotherapy (Tomo) mimic plan. (d) Comparison of all the mimic plans. All mimic plans achieved good agreement of the target and organ at risk dose-volume histograms. *Abbreviations:* PTV SR = planning target volume standard risk; PTV HR = planning target volume high risk; Lt = Left; Rt = Right

For the tomotherapy mimic plans, only the spinal cord maximum dose was clinically different (>5%), although in this case, the dose was lower in the deliverable plan than the predicted plan. Seven of the 9 clinical goals (maximum dose to external and cord, mean dose and D50 to right and left parotids, and mean larynx dose) were statistically significantly different (*P* < .03) than the predicted dose. However, except for the external maximum dose, all the OAR doses were lower in the deliverable tomotherapy plans than the predicted dose. Figure 3c shows the distribution of the percent difference between the predicted doses and mimicked tomotherapy plans for select dosimetric values. A full comparison can be found in Table E5. Figure 4 shows the DVH, a common plot for assessing RT plans that shows the volume of different organs or targets that receive a given dose or above for a representative patient, demonstrating the similarity in the dose distribution between the predicted and mimicked dose distributions.

### Across protocol

**Figure 5:**
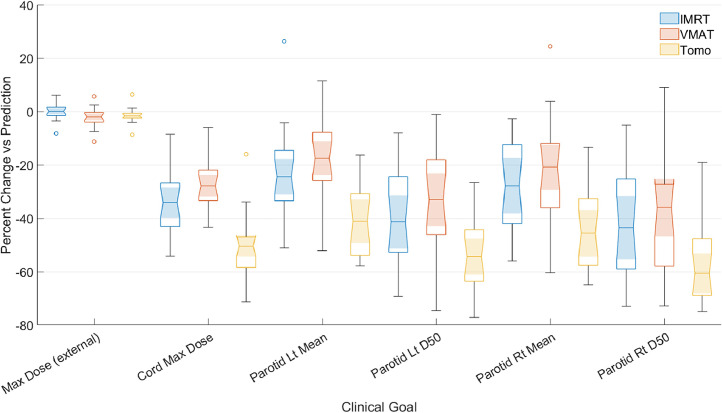
Comparison of multicriteria optimizer (MCO) and predicted dose distributions. Percent change of the dosimetric values obtained for select clinical goals between the predicted dose distributions and MCO plans. The colors correspond to the treatment technique used in the MCO plan, and the shaded region indicates the 95% CI around the median. *Abbreviations:* IMRT = intensity modulated radiation therapy; Tomo = tomotherapy; Lt = Left; Rt = Right; D50 = Dose delivered to 50% of volume.

**Figure 6:**
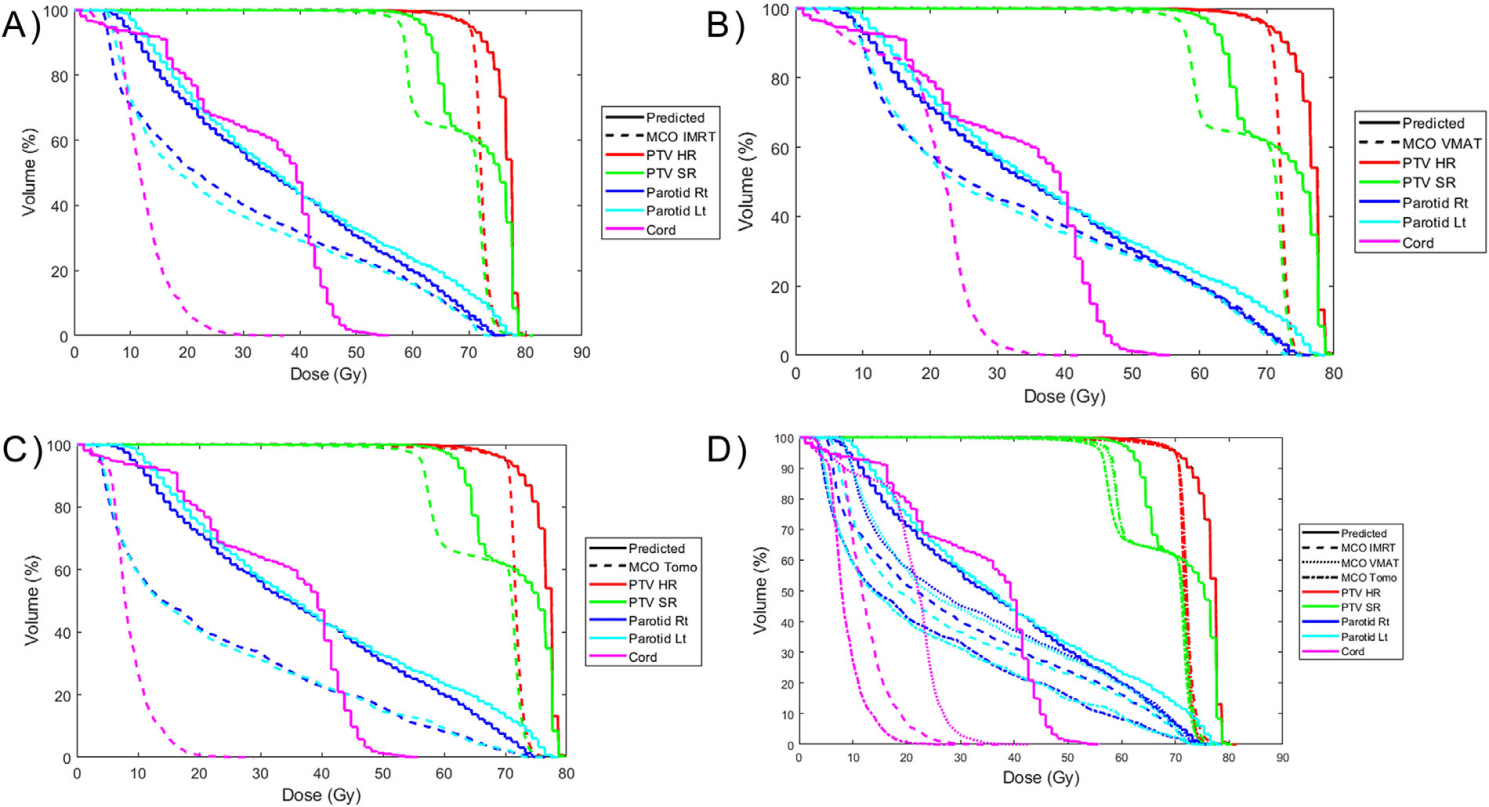
Comparison of the dose-volume histograms for a representative case multicriteria optimizer (MCO) plans. (a) Comparison of the predicted dose with the intensity modulated radiation therapy (IMRT) MCO plan. (b) Comparison with the volumetric modulated arc therapy (VMAT) MCO plan. (c) Comparison with the tomotherapy (Tomo) MCO plan. (d) Comparison of all the MCO plans with the predicted dose. In all cases, the uniformity of the planning target volume (PTV) doses at the prescription dose was increased, allowing for a lower total dose and substantial reduction in the dose to the parotids and the spinal cord. *Abbreviations:* PTV SR = planning target volume standard risk; PTV HR = planning target volume high risk..

The integration of the MCO into the clinical plan creation process was found to greatly decrease the dose of OARs relevant to the clinical goals used at our institution. Although the average PTV standard risk coverage was lower, on average, in the MCO-integrated plans, it remained >95% for all the cases investigated, because this is the clinical coverage standard used to generate the optimization functions. The difference in clinical goals was found to be clinically relevant (>5%) and statistically (*P* < .03) significant in all cases except for the maximum dose to the external and the larynx mean dose. Figure 5 shows the distribution of the percent change of the dosimetric values obtained for select clinical goals between MCO plans and the predicted dose distributions. Figure 6 shows comparisons of the IMRT, VMAT, and tomotherapy MCO plan DVHs to the prediction. Among the 3 MCO plans, there was no clinically relevant difference between the IMRT MCO and VMAT MCO plans as assessed by the clinical goals. However, the cord (maximum) and parotid (mean and D50) doses were significantly lower in the tomotherapy MCO plans compared with the VMAT MCO plans (*P* < .05). The dose to these structures was, on average, lower in the tomotherapy MCO plans compared with the IMRT MCO plans, but the difference did not meet the level of significance (*P* > .05).

### Constrained versus unconstrained MCO

Optimizing using only the generic MCO objectives, without using the prediction-based constraints, gave similar “balanced” plans where all Pareto plans were weighted equally, as the prediction-informed MCO optimization. Although there were differences in the individual plans, we found no significant differences in the clinical goals overall between the plans optimized using the prediction-constrained and -unconstrained MCO. Again, to keep a standardized workflow, the “balanced” MCO result was used to generate the final deliverable plan. Using this method, the pure optimization time (to generate the Pareto plans and deliverable segments, not including any of the setup steps) decreased by an average of 2 minutes when the constraints were removed (from an average of 8 minutes to an average of 6 minutes). Including the import and setup steps, the total prediction-to-plan time was approximately 20 minutes.

### DQA testing

DQA for all cases passed above 98% (range, 98%-100%) using a gamma criterion of 3%/2 mm, well above the 90% threshold recommendation by the American Association of Physicists in Medicine.^41^ As such, the mimicked and MCO plans for all 3 modalities were considered clinically deliverable.

## Discussion

We successfully developed a workflow using dose-mimicking optimization to rapidly, within 20 minutes, create deliverable plans matching the dose distributions predicted by our model, using both the same modality as in the plans used for training the model, IMRT, as well as for different modalities, VMAT and tomotherapy. Based on the clinical goals, the predicted dose distributions produced by our model are deliverable and clinically acceptable across all modalities investigated. The noted difference in the external and cord maximum dose between the predicted doses and the mimicked plans is likely explained by the optimization process; as the mimic dose operation attempts to match the DVH and the voxel-by-voxel dose of the predicted dose, it is understandable that the maximum dose, which is defined by both a small portion of the DVH and a small number of the voxels, could be missed in the optimization process. We found that the inclusion of maximum dose optimization objectives for the cord and external based on the prediction reinforced the importance of these metrics in the optimizer and reduced the difference of the maximum dose to these structures below clinically relevant levels (<5%) while having little to no effect on the other clinical goals. As such, we would recommend explicitly adding optimization objectives based on the prediction for important, small-volume dose points. More importantly, because the predicted dose distributions are clinically accurate across a variety of treatment modalities, it is potentially feasible to create a single trained model that could be used for plan generation across multiple treatment modalities.

Integrating the MCO into the workflow further allowed quick adjustments to reflect institutional preference, opening the possibility of extending a single model across multiple institutions. Additionally, this makes it easier to tailor a dose distribution to edge cases that would not be well captured in the training set, such as patients with prior radiation that have lower than usual dose tolerance to certain OARs. Although it is a powerful tool, the user still needs to define proper objectives and constraints to ensure the Pareto surface covers the full area of potential interest while restricting it to only clinically acceptable plans to increase the ease of arriving at a high-quality plan.^42^ Our model serves as a useful way to assist with these definitions. Although in this work, only the parotid D50 and the external and spinal cord maximum doses were set as hard constraints, it would be possible to set hard constraints for all OARs of interest, although the dose constraints used would likely have to be slightly raised to give some room to modify the dose distribution.

However, our work on the MCO approach did show that a prediction model is less accurate when the current plan and training plans have differing dosimetric preferences or if the training set is not composed of well-optimized plans, as seen by the significant difference in most of the clinical goals seen in section 3.4. In our study, we observed average reductions nearing 45% for certain structures when comparing the MCO result with the initial test plan based on predictions, specifically for dose metrics that hold significance at our institution (refer to Table E6). In addition, we found no significant difference between plans optimized where the MCO had constraints set using the predicted dose distribution versus those optimized using only the generic MCO template when using the balanced MCO result. This implies that the predicted values were too far above the achievable values to meaningfully impact the optimization process. This is a common problem with supervised learning models, because the trained model will not deviate far from the data on which it was trained. As such, it is vital that any institution using such a model, including the one proposed in this work, perform its own independent validation of the model's performance prior to using it clinically. However, because all of the training plans were considered “clinically acceptable,” the resulting predictions could still help guide a less experienced planner in navigating the MCO. For example, should the planner want to further reduce the parotid dose at the expense of the spinal cord, they could use the prediction to assess how high they could allow the cord dose to get for a particular patient based on previous plans. The baseline provided by the prediction could, therefore, provide a useful reference point for the planner and the physician performing the final plan assessment. As with other supervised learning models, these MCO-generated plans could be incorporated into the training set to iteratively improve the performance of the model by further increasing the quality of the training plans and/or including more plans that match an institution's specific preference.

Additionally, our dose prediction model was unable to predict dose distributions directly for plans with multiple targets where the target prescription ratios are different from those used in the training set. Although changes in the overall scaling can be handled by simply renormalizing the isodose distribution to the desired prescription, if the ratio between target doses changes, then the isodose distribution must be adjusted, most significantly in the gradient between targets. For example, for the dose falloff from the edge of the PTV_HR into the PTV standard risk, the dose difference in the prescription determines how much the dose needs to be reduced at that interface, whereas at the edge of the target into the normal tissue, the dose reduction goal is always the same (as low as reasonably achievable) with the gradient determined by the proximity and dose tolerance of surrounding OARs.

Recently developed reinforcement learning approaches^43^, ^44^, ^45^ offer one alternative that can allow for a way to reduce the overhead involved in transferring a model between institutions. The reward function used to assess the plans can be modified to suit the specific preference of an institution, and relatively few patient cases (10s instead of 100s) are needed for training. However, a new model does need to be trained each time the reward function is modified, which would slow deployment at a new center. Further, the current architectures do not scale well with the number of relevant OARs (of which there are many for head and neck cases), although work is being done to minimize this issue.^43^

Future work will focus on methods to allow for the model to be applicable across multiple prescription levels, which would extend the flexibility of the model. Further work could also be done to develop an efficient method to modify a standardized trained model to conform to the specific preference of an institution. This can help deploy individualized models to medical centers without sufficient resources to build a model on their own.

## Conclusions

Our deep learning-based model can create predicted dose distributions that are deliverable with a variety of RT treatment modalities, including those not used to create the plans used for training the model. Integrating the MCO allows for customization of the predicted plan with only a minor increase in the planning time.

## Appendix Supplementary materials

Auto_Planning_For_Radiotherapy_SupplementTables_new Image, application 1

## Footnotes

Supplementary material associated with this article can be found in the online version at doi:10.1016/j.adro.2024.101649.
