# Supplementary material for: Automatic Treatment Planning for Radiation Therapy: A Cross-Modality and Protocol Study
Source: Adv Radiat Oncol. 2024 Oct 9;9(12):101649. doi: 10.1016/j.adro.2024.101649 (PMC11566342; doi:10.1016/j.adro.2024.101649)
Supplement: Auto_Planning_For_Radiotherapy_SupplementTables_new [file mmc1.docx]

# Supplementary Data

| **Supplementary Table 1:** Comparison of predicted vs mimicked dose distributions using IMRT | | | |
| --- | --- | --- | --- |
| **Clinical Goal** | **Mimicked-Predicted** | **% difference** | **p-value** |
| PTV_SR Coverage | -0.12 ± 0.37 % | -0.12 ± 0.39 | 0.43 |
| External Max Dose | 0.8 ± 1.0 Gy | 1.11 ± 1.25 | 0.00* |
| Cord Max Dose | 2.1 ± 2.7 Gy | 5.57 ± 6.97 | 0.00* |
| Lt Parotid Mean | 0.0 ± 0.9 Gy | 0.42 ± 4.08 | 0.77 |
| Lt Parotid D50 | 0.1 ± 1.5 Gy | 1.58 ± 6.03 | 0.62 |
| Rt Parotid Mean | 0.1 ± 0.7 Gy | 0.45 ± 1.93 | 0.22 |
| Rt Parotid D50 | -0.6 ± 1.0 Gy | -1.52 ± 3.11 | 0.05 |
| Larynx Mean | -0.4 ± 1.2 Gy | -1.69 ± 3.84 | 0.30 |
| Larynx V60 | 1.13 ± 1.08 % | 5.88 ± 7.97 | 0.38 |
|  | *statistically significant, *p*<0.05 | | |

| **Supplementary Table 2:** Comparison of predicted vs mimicked dose distributions using VMAT | | | |
| --- | --- | --- | --- |
| **Clinical Goal** | **Mimicked-Predicted** | **% difference** | **p-value** |
| PTV_SR Coverage | 0.04 ± 0.51 % | 0.04 ± 0.53 | 0.21 |
| External Max Dose | 0.7 ± 1.3 Gy | 0.89 ± 1.67 | 0.00* |
| Cord Max Dose | 1.0 ± 1.7 Gy | 2.83 ± 4.84 | 0.04* |
| Lt Parotid Mean | 0.0 ± 0.8 Gy | 0.68 ± 2.92 | 0.49 |
| Lt Parotid D50 | 0.0 ± 1.1 Gy | 1.49 ± 5.95 | 0.99 |
| Rt Parotid Mean | 0.1 ± 0.8 Gy | 0.55 ± 2.08 | 0.38 |
| Rt Parotid D50 | -0.2 ± 1.0 Gy | -0.16 ± 2.39 | 0.83 |
| Larynx Mean | -0.3 ± 1.4 Gy | -1.53 ± 4.64 | 0.91 |
| Larynx V60 | -0.27 ± 0.85 % | -2.23 ± 9.98 | 0.33 |
|  | *statistically significant, *p*<0.05 | | |

| **Supplementary Table 3:** Comparison of predicted vs mimicked dose distributions using Tomotherapy | | | | | | | |  |  |
| --- | --- | --- | --- | --- | --- | --- | --- | --- | --- |
| **Clinical Goal** | | **Mimicked-Predicted** | | **% difference** | | **p-value** | |  |  |
| PTV_SR Coverage | | 0.06 ± 0.33 % | | 0.06 ± 0.34 | | 0.33 | |  |  |
| External Max Dose | | 0.6± 1.4 Gy | | 0.72 ± 1.84 | | 0.03* | |  |  |
| Cord Max Dose | | -2.6 ± 1.8 Gy | | -6.29 ± 4.12 | | 0.00* | |  |  |
| Lt Parotid Mean | | -0.5 ± 0.8 Gy | | -1.24 ± 1.46 | | 0.01* | |  |  |
| Lt Parotid D50 | | -0.6 ± 0.9 Gy | | -1.74 ± 2.86 | | 0.02* | |  |  |
| Rt Parotid Mean | | -0.7 ± 0.7 Gy | | -1.62 ± 1.53 | | 0.01* | |  |  |
| Rt Parotid D50 | | -1.1 ± 1.2 cGy | | -2.44 ± 2.24 | | 0.03* | |  |  |
| Larynx Mean | | -1.4 ± 1.0 Gy | | -2.89 ± 2.35 | | 0.02* | |  |  |
| Larynx V60 | | -1.23 ± 1.93% | | -9.35 ± 24.46 | | 0.38 | |  |  |
|  | | *statistically significant, *p*<0.05 | | | | | |  |  |
| **Supplementary Table 4:** Summary comparison of predicted vs mimicked dose distributions for various modalities | | | | | | | | | |
|  | | **IMRT** | | **VMAT** | | | **Tomotherapy** | | |
| **Clinical Goal** | | **% difference** | **p-value** | **% difference** | | **p-value** | **% difference** | **p-value** | |
| PTV_SR Coverage | | -0.12 ± 0.39 | 0.43 | 0.04 ± 0.53 | | 0.21 | 0.06 ± 0.34 | 0.33 | |
| External Max Dose | | 1.11 ± 1.25 | 0.00* | 0.89 ± 1.67 | | 0.00* | 0.72 ± 1.84 | 0.03* | |
| Cord Max Dose | | 5.57 ± 6.97 | 0.00* | 2.83 ± 4.84 | | 0.04* | -6.29 ± 4.12 | 0.00* | |
| Lt Parotid Mean | | 0.42 ± 4.08 | 0.77 | 0.68 ± 2.92 | | 0.49 | -1.24 ± 1.46 | 0.01* | |
| Lt Parotid D50 | | 1.58 ± 6.03 | 0.62 | 1.49 ± 5.95 | | 0.99 | -1.74 ± 2.86 | 0.02* | |
| Rt Parotid Mean | | 0.45 ± 1.93 | 0.22 | 0.55 ± 2.08 | | 0.38 | -1.62 ± 1.53 | 0.01* | |
| Rt Parotid D50 | | -1.52 ± 3.11 | 0.05 | -0.16 ± 2.39 | | 0.83 | -2.44 ± 2.24 | 0.03* | |
| Larynx Mean | | -1.69 ± 3.84 | 0.30 | -1.53 ± 4.64 | | 0.91 | -2.89 ± 2.35 | 0.02* | |
| Larynx V60 | | 5.88 ± 7.97 | 0.38 | 7.18 ± 8.50 | | 0.33 | -9.35 ± 24.46 | 0.38 | |
|  | | *statistically significant, *p*<0.05 | | | | | | | |

| **Supplementary Table 5:** Comparison of predicted dose vs plans created using MCO method | | | | | | |
| --- | --- | --- | --- | --- | --- | --- |
|  | **IMRT** | | **VMAT** | | **Tomotherapy** | |
| **Clinical Goal** | **Difference** | **% difference** | **Difference** | **% difference** | **Difference** | **% difference** |
| PTV_SR Coverage | -1.1 ± 1.0 % | -1.1 ± 1.0 | -0.8 ± 1.1 % | -0.8 ± 1.1 | -2.5 ± 1.0 % | -2.6 ± 1.0 |
| External Max Dose | 0.0 ± 2.5 Gy | 0.1 ± 3.1 | -1.7 ± 3.0 Gy | -2.1 ± 3.6 | -1.2 ± 2.2 Gy | -1.5 ± 2.7 |
| Cord Max Dose | -14.0 ± 6.3 Gy | -33.0 ± 12.5 | -11.9 ± 5.9 Gy | -28.2 ± 11.4 | -21.4 ± 6.7 Gy | -50.8 ± 11.4 |
| Lt Parotid Mean | -8.1 ± 4.6 Gy | -23.4 ± 16.7 | -6.6 ± 5.1 Gy | -18.5 ± 15.0 | -14.0 ± 4.8 Gy | -40.3 ± 12.5 |
| Lt Parotid D50 | -13 ± 6.2 Gy | -39.7 ± 18.0 | -12.1 ± 7.9 Gy | -34.2 ± 19.2 | -18.0 ± 7.2 Gy | -52.9 ± 13.4 |
| Rt Parotid Mean | -9.3 ± 5.1 Gy | -28.0 ± 16.2 | -7.7 ± 6.0 Gy | -22.2 ± 19.2 | -15 ± 4.8 Gy | -44.2 ± 14.8 |
| Rt Parotid D50 | -13.6 ± 5.6 Gy | -43.1 ± 18.5 | -12.5 ± 6.8 Gy | -37.8 ± 20.7 | -18.6 ± 6.1 Gy | -56.6 ± 14.2 |
| Larynx Mean | -3.3 ± 8.8 Gy | -6.9 ± 19.2 | -6.0 ± 8.2 Gy | -10.9 ± 16.0 | -7.6 ± 10.7 Gy | -15.0 ± 22.5 |
| Larynx V60 | 1.7 ± 14.7 % | 22.3 ± 165.0 | -6.2 ± 11.0 % | 35.0 ± 140.0 | -3.0 ± 14.5 % | -17.7 ± 125.3 |

| **Supplementary Table 6**: Tradeoff constraints and objective used for MCO integration. The objectives were set to be unrealistically low to push as hard as possible. Values marked with “X” are pulled from the dose prediction | |
| --- | --- |
| ROI | Goal |
| Constraints | |
| High Risk PTV | Volume receiving 70 Gy ≥ 95% |
| Intermediate Risk PTV* | Volume receiving 63 Gy ≥ 95% |
| Standard Risk PTV | Volume receiving 56 Gy ≥ 95% |
| Spinal Cord | Max: X Gy point dose |
| External | Max: X Gy point dose |
| Parotid Rt/Lt | 50% receives < X Gy |
| Objectives | |
| High Risk PTV | Uniform dose 70 Gy |
|  | Volume receiving 70 Gy = 100% |
| Intermediate Risk PTV* | Volume receiving 63 Gy = 100% |
| Standard Risk PTV | Volume receiving 56 Gy = 100% |
| Intermediate Risk PTV – High Risk PTV* | Max: 63 Gy point dose |
| Standard Risk PTV – High Risk PTV | Max: 56 Gy point dose |
| External | Dose fall-off: 56 Gy to 46 Gy in 0.5 cm |
|  | Dose fall-off 56 Gy to 5 Gy in 5 cm |
|  | Max: 70.5 Gy point dose |
| Parotid Rt/Lt | Max EUD: 1 Gy, A = 1 |
| Spinal Cord | Max: 5 Gy point dose |
| Larynx* | Max EUD: 10 Gy A = 1 |
|  | Max: 50 Gy point dose |
| Esophagus* | Volume receiving 1 Gy < 33% |
|  | Volume receiving 1 Gy < 66% |
| *When applicable | |

| **Supplementary Table 7**: Target and OAR constraints used at our institution for head and neck planning | |
| --- | --- |
| ROI | Clinical Goal |
| High Risk PTV | Volume receiving 70 Gy ≥ 95% |
| Intermediate Risk PTV* | Volume receiving 63 Gy ≥ 95% |
| Standard Risk PTV* | Volume receiving 56 Gy ≥ 95% |
| Spinal Cord | Max: 50 Gy to point dose |
| Parotid Rt/Lt | Mean dose < 26 Gy |
|  | 50% receives < 30 Gy |
| Larynx | Mean dose ≤ 41 Gy |
|  | Volume receiving 60 Gy ≤ 24% |
| External (all tissue) | Max: 77 Gy to point dose |
| *When applicable | |
